# Whole Blood RNA as a Source of Transcript-Based Nutrition- and Metabolic Health-Related Biomarkers

**DOI:** 10.1371/journal.pone.0155361

**Published:** 2016-05-10

**Authors:** Petar D. Petrov, M. Luisa Bonet, Bárbara Reynés, Paula Oliver, Andreu Palou, Joan Ribot

**Affiliations:** Laboratory of Molecular Biology, Nutrition and Biotechnology-Nutrigenomics, University of the Balearic Islands and CIBER Fisiopatología de la Obesidad y Nutrición (CIBERobn), Palma de Mallorca, Spain; University of Kansas Medical Center, UNITED STATES

## Abstract

Blood cells are receiving an increasing attention as an easily accessible source of transcript-based biomarkers. We studied the feasibility of using mouse whole blood RNA in this context. Several paradigms were studied: (i) metabolism-related transcripts known to be affected in rat tissues and peripheral blood mononuclear cells (PBMC) by fasting and upon the development of high fat diet (HFD)-induced overweight were assessed in whole blood RNA of fasted rats and mice and of HFD-fed mice; (ii) retinoic acid (RA)-responsive genes in tissues were assessed in whole blood RNA of control and RA-treated mice; (iii) lipid metabolism-related transcripts previously identified in PBMC as potential biomarkers of metabolic health in a rat model were assessed in whole blood in an independent model, namely retinoblastoma haploinsufficient (Rb^+/-^) mice. Blood was collected and stored in RNAlater^®^ at -80°C until analysis of selected transcripts by real-time RT-PCR. Comparable changes with fasting were detected in the expression of lipid metabolism-related genes when RNA from either PBMC or whole blood of rats or mice was used. HFD-induced excess body weight and fat mass associated with expected changes in the expression of metabolism-related genes in whole blood of mice. Changes in gene expression in whole blood of RA-treated mice reproduced known transcriptional actions of RA in hepatocytes and adipocytes. Reduced expression of Fasn, Lrp1, Rxrb and Sorl1 could be validated as early biomarkers of metabolic health in young Rb^+/-^ mice using whole blood RNA. Altogether, these results support the use of whole blood RNA in studies aimed at identifying blood transcript-based biomarkers of nutritional/metabolic status or metabolic health. Results also support reduced expression of Fasn, Lrp1, Rxrb and Sorl1 in blood cells at young age as potential biomarkers of metabolic robustness.

## Introduction

Peripheral blood mononuclear cells (PBMC) constitute an easily obtainable fraction of blood cells (less than 0.1% of the total blood cells) consisting basically in lymphocytes and monocytes [1]. Studies using microarray analysis have shown that PBMC express a large proportion (approximately 80%) of the genes encoded in the human genome and that gene expression in these cells responds to internal and external signals and reflects metabolic and physiological adaptations, thus providing a large biosensor pool in the form of gene transcripts [2, 3]. PBMC have been proven useful in metabolic phenotyping studies, as they can reflect, for instance, the effects of specific diets and feeding conditions (such as fasting/refeeding) on gene expression that occur in key tissues in energy homeostasis, such as liver and white adipose tissue (WAT) [4–8] or the effects of environmental stresses (such as cold acclimation) on gene expression that occur in brown adipose tissue (BAT) [9]. PBMC are also of interest for clinical diagnostic purposes, since they can reflect gene expression patterns of different pathologies [10, 11], and have been proposed as a source of biomarkers of obesity [5, 7, 12, 13] or altered (increased or decreased) predisposition to obesity [14, 15]. Of particular interest is the identification of early transcript-related biomarkers that, before the onset of disease, specifically identify predisposition or predict the individual capacity to deal with environmental (including dietary) and age-related stresses [16].

Mouse is the most used animal model and there is extensive literature on the use of mouse models for drug testing and biomarker discovery [17]. Surprisingly, the use of mouse PBMC as a source of biomarkers is quite limited and only few studies report it (e.g. [18]). This situation probably has to do with the limited volume of blood that can be taken safely in a single extraction in mice, much lower than the blood volume necessary for obtaining the PBMC fraction, which in mice is only achievable using terminal procedures such as cardiac puncture. Moreover, PBMC isolation is a relatively labour intensive process that requires technical expertise at the time of sample collection, and PBMC pellets, unlike RNA-stabilized whole blood samples, cannot be stored for prolonged time periods as this provokes substantial changes in the gene expression profile [19]. This scenario has prompted the use of whole blood, which has been claimed might be an ideal tissue for the identification of novel genomic biomarkers, especially when only minimal volumes of blood can be obtained, as when working with small animals [20, 21].

Transcriptional analysis of mouse whole blood RNA, rather than PBMC RNA, could therefore be a better choice for the identification of transcript-based biomarkers in mice as part of the preclinical validation of human biomarkers. Here, we aimed to establish the usefulness of whole blood transcriptional analysis in mice as a tool in studies dealing with metabolic and physiological adaptations in front of nutritional and pharmacological interventions and to validate previously reported early blood cells transcript-based biomarkers of metabolic health. To these ends, several experimental paradigms were studied. First, transcript levels of metabolism-related genes known to be affected in rat PBMC by fasting [7, 22] and upon high fat diet (HFD)-induced overweight [12, 23] were assessed in whole blood RNA from fasted rats and mice and from HFD-fed mice. Second, transcript levels of known retinoic acid (RA)-responsive genes in tissues of mice (see [24, 25]) were assessed in whole blood RNA of control and RA-treated mice. Finally, a set of lipid metabolism-related transcripts previously identified from PBMC RNA as putative biomarkers of metabolic robustness in a rat model [14] were assessed in a mouse model of metabolic robustness, namely retinoblastoma (Rb) haploinsufficient mice [26, 27], using whole blood RNA.

## Material and Methods

### Animals and study designs

All animal protocols were in accordance with the national and European ethical guidelines for the use and care of laboratory animals and were approved by the Bioethical Committee of the University of the Balearic Islands. In all the experiments, animals were housed in plastic cages at 22°C with a period of light/dark of 12 h (lights on from 08:00 to 20:00 hours).

#### Fasting experiments

Two and a half-month-old male Wistar rats (Charles River Laboratories, Barcelona, Spain) housed individually and fed with a standard chow diet (Panlab, Barcelona, Spain) were used. The animals were distributed into two groups (3 animals per group): a control-fed group, animals provided with *ad libitum* access to chow diet, and a fasted group, animals deprived of food for 12 h (from 20:00 to 08:00h). Coprophagy was prevented by changing the cage immediately prior to food deprivation. At the beginning of the light cycle, animals under *ad libitum* or fasting conditions were weighed and blood was collected from the saphena vein. Part of the blood (~70 μL) was collected in heparinized capillary tubes and used to obtain plasma, another part (~2mL) was mixed with EDTA and used to obtain the PBMC fraction (see below), and a third part (~200 μL) was mixed with *RNAlater*^*®*^ (~1:5; Ambion^®^, Life Technologies, Alcobendas, Spain) to quickly stabilize the RNA and stored at -80°C for later whole blood RNA isolation and gene expression analysis. In parallel, an experiment with 2.5-month-old NMRI male mice (Charles River Laboratories) was conducted with the difference that mice were deprived of food for just 8h (from 24:00 to 08:00h) and blood was collected from the submandibular vein. One part of the blood (~70 μL) was collected with heparinized capillary tubes to obtain plasma, and another part (~200 μL) was mixed with *RNAlater*^*®*^ (~1:5; Ambion^®^, Life Technologies) and stored at -80°C for later RNA isolation and gene expression analysis. The experiments using male Wistar rats and NMRI mice were performed twice.

A separate fasting experiment was conducted with 3-month-old CD-1 female mice (own production) housed 2–3 mice per cage under the standard conditions indicated above and distributed into a control-fed group and a fasted group deprived of food for 12 h (from 20:00 to 08:00h) (5–6 animals per group). At the beginning of the light cycle, both groups of animals were anaesthetized by intraperitoneal injection of ketamine (100 mg/kg body weight) and xylazine (10 mg/kg body weight) and blood was collected by cardiac puncture (open approach) using a 23 G needle attached to a 1 mL syringe with EDTA. Part of the blood (~70 μL) was used to obtain plasma, another part (~750 μL) was used to obtain the PBMC fraction (see below), and a third part (~200 μL) was mixed with *RNAlater*^*®*^ (~1:5; Ambion^®^, Life Technologies) and stored at -80°C for later RNA isolation and gene expression analysis.

#### HFD experiment

One and a half-month-old C57BL/6J male mice (Charles River Laboratories) housed 2–3 mice per cage were used. Mice (at least 6 animals per group) were fed *ad libitum* with a defined low fat diet (D12450J; Research Diets Inc., New Brunswick, NJ; 10% energy as fat; LF group) or a defined high fat diet (D12451; Research Diets Inc.; 45% energy as fat; HF group). Body weight and body composition using EchoMRI-700™ analyzer (Houston, TX) were assessed the day before blood extraction. At the beginning of the light cycle, whole blood was collected from the submandibular vein (~150 μL), mixed with *RNAlater*^*®*^ (~1:5; Ambion^®^, Life Technologies) and stored at -80°C for later RNA isolation and gene expression analysis.

#### RA experiment

Three-month-old NMRI male mice (Charles River Laboratories) housed 2–3 mice and fed with a standard chow diet (Panlab; 2.5 IU vitamin A/kcal) were used. Mice received one daily subcutaneous injection of all-*trans* RA (ATRA; Sigma-Aldrich, St. Louis, MO) at a dose of 50 mg/kg body weight or the vehicle (100 μL olive oil) during the 4 days before euthanization (5–6 animals per group). Body weight and energy intake were followed daily during the treatment period. Energy intake was estimated on a per-cage basis from the actual amount of food consumed by the animals. The animals were killed with CO_2_ and decapitated at the start of the light cycle. Blood was collected from the neck and serum prepared by centrifugation and frozen at -80°C until biochemical analysis. A part of the blood (~200 μL) was mixed with *RNAlater*^*®*^ (~1:5; Ambion^®^, Life Technologies) and stored at -80°C for later RNA isolation and gene expression analysis. Epididymal WAT (eWAT), liver and gastrocnemius skeletal muscle were excised in their integrity and weighted. The weight of the eWAT depot expressed as percentage of body weight was previously shown to largely correlate with total body fat content [28] and was used as adiposity index.

#### Rb haploinsufficient mice experiment

Wild-type (WT, Rb^+/+^) and Rb1tm1Tyj (Rb^+/−^) C57BL/6J male mice (originally purchased from the Jackson Laboratory–Bar Harbor, ME–and maintained in the University of the Balearic Islands animal house) housed 2–3 mice per cage and fed with 5K67 diet (LabDiet, St. Louis, MO) were used. Rb^+/−^ and WT mice were studied at two, five and seven months of age. For each age, at the beginning of the light cycle, mice (at least 6 animals per genotype) were weighed and blood was collected (~150 μL) from the submandibular vein, mixed with *RNAlater*^*®*^ (~1:5; Ambion^®^, Life Technologies) and stored at -80°C for later RNA isolation and gene expression analysis. Body composition was determined using an EchoMRI-700™ analyzer two days before blood extraction.

### PBMC isolation

Fresh blood samples of fed and 12h-fasted rats and mice were used to isolate PBMC using OptiPrep^TM^ density-gradient separation (60% (w/v) solution of iodixanol in water; Sigma-Aldrich) according to the manufacturer's instructions. Briefly, peripheral blood samples collected using EDTA (final concentration of 3–4 mM) as anticoagulant were diluted up to 6 mL with buffered saline (0.85% (w/v) NaCl, 10 mM Hepes at pH 7.4). The diluted blood was layered carefully on to 3 mL of a 1.077 g/mL density barrier prepared by diluting 2.7 vol of OptiPrep^TM^ reagent in 9.3 vol. of diluted buffered saline (2.5:0.5, v/v in water) and centrifuged at 700 *g* for 20 min at 20 °C in a swinging-bucket rotor with acceleration and deceleration at zero. PBMC were harvested from the sharp band at the interface, diluted with 2 vol. of buffered saline and pelleted at 400 *g* for 10 min at 20 °C for later RNA isolation and gene expression analysis.

### RNA isolation and gene expression analysis

Total RNA was extracted from blood samples stored in *RNAlater*^*®*^ using Mouse RiboPure™-Blood RNA Isolation Kit (Ambion^*®*^, Life Technologies) and from PBMC using E.Z.N.A.^*®*^ total RNA kit I (Omega Bio-Tek, Norcross, GA), following the manufacturer’s instructions. RNA was further purified by standard ethanol/sodium acetate precipitation. The concentration and purity of RNA were estimated using a Nano-Drop 1000 instrument (Thermo-Fisher Scientific, Waltham, MA). RNA was analyzed for integrity on a 1% agarose gel stained with SYBRSafe (Life Technologies, Carlsbad, CA) and stored at −80°C until analysis. Total RNA (50 ng/reaction) was retrotranscribed using an iScript cDNA synthesis kit (Bio-Rad, Madrid, Spain), using random hexamers priming. mRNA expression levels of genes of interest were analyzed by real-time PCR using the StepOnePlus system with SYBR Green I sequence nonspecific detection (Life Technologies), essentially as described before [26] and following the MIQE guidelines [29]. For each reaction, Power SYBR Green Master mix (Life Technologies), 200 nM of forward and reverse primers, and 2.5 ng cDNA equivalents were used. Following amplification, melt curve analysis was performed in each plate. The sequences of the primer sets used are available upon request. The identity of the amplicons was confirmed by resolving the PCR reaction products on 2–4% agarose gels and by comparing the observed melting temperature with the predicted one (by Poland algorithm; see [30]). Raw amplification data (Rn) were exported and used to determine the efficiency of the amplification and the cycle of quantification by the LinRegPCR software [31]. Relative gene expression was calculated using the Pfaffl method, correcting for the different efficiencies [32].

### Circulating parameters

Blood glucose concentration was measured with an Accu-Chek Glucometer (Roche Diagnostics, Barcelona, Spain). Plasma or serum non-esterified fatty acid (NEFA) and β-hydroxybutyrate concentrations were measured using commercial enzymatic colorimetric kits (from, respectively, Wako Chemicals, Neuss, Germany; and Ben S.r.l. Biochemical Enterprise, Milano, Italy) according to the manufacturer’s instructions.

### Statistical analysis

All data are expressed as the mean ± SEM. Differences between groups were assessed by Student’s *t* test. In the correlation analyses, significant correlations were assessed by Pearson's coefficients. Analyses were performed with SPSS^*®*^ software for Windows^*®*^ (IBM España, Madrid, Spain) and threshold of significance was defined at p<0.05.

## Results and Discussion

### Whole blood can reflect transcriptional responses to fasting as PBMC do

Several adaptations occur during fasting to maintain energy homeostasis and liver and WAT play crucial roles in this metabolic response. Liver glycogen serves as a fuel in the first hours of food deprivation, after which mobilization of gluconeogenic precursors and of alternative fuels occur, together with an increase in hepatic gluconeogenesis to maintain a constant supply of glucose to the brain (see [33, 34]). Breakdown of triacylglycerols in WAT and partial oxidation of fatty acids (FAs) in the liver lead to the production of NEFA and ketone bodies that enter the bloodstream and can be used as energy sources by other tissues (see [35]). As shown in Table 1, we could reproduce in food-deprived rats and mice expected changes in blood glucose, NEFA and β-hydroxybutyrate levels. These and other adaptations have been associated with coordinated changes in the expression of key genes in liver and adipose tissue that allow to reprogram the metabolic pathways to face the situation of food deprivation [36]. In particular, a rapid decrease in the expression of lipogenic genes followed by an induction of lipolytic and FA oxidation-related genes, which occurs first in liver and afterwards in WAT, has been described following food deprivation [36].

**Table 1:** Body weight, blood glucose concentration and plasma concentration of non-esterified fatty acids (NEFA) and β-hydroxybutyrate in *ad libitum* fed and fasted animals.

	Wistar male rats		CD1 female mice		NMRI male mice	
	fed	12h fasted	fed	12h fasted	fed	8h fasted
body weight (g)	347±9.8	310±4.2*	32.1±0.5	29.3±1.0*	41.8±1.4	37.9±0.9*
glucose (mM)	5.59±0.34	4.56±0.12*	14.3±0.47	10.0±0.42*	8.68±0.29	7.80±0.26*
NEFA (mM)	0.39±0.05	0.70±0.08*	0.24±0.02	0.45±0.06*	0.68±0.08	1.20±0.06*
β-hydroxybutyrate (mM)	0.43±0.11	0.90±0.15*	0.58±0.54	5.40±0.70*	0.30±0.12	0.91±0.16*

Data are means ± SEM of 4–6 animals per group.

* Indicates that mean values in the fasted group and the fed group are statistically different (P<0.05; Two-tailed Student’s t test)

**Fig 1:**
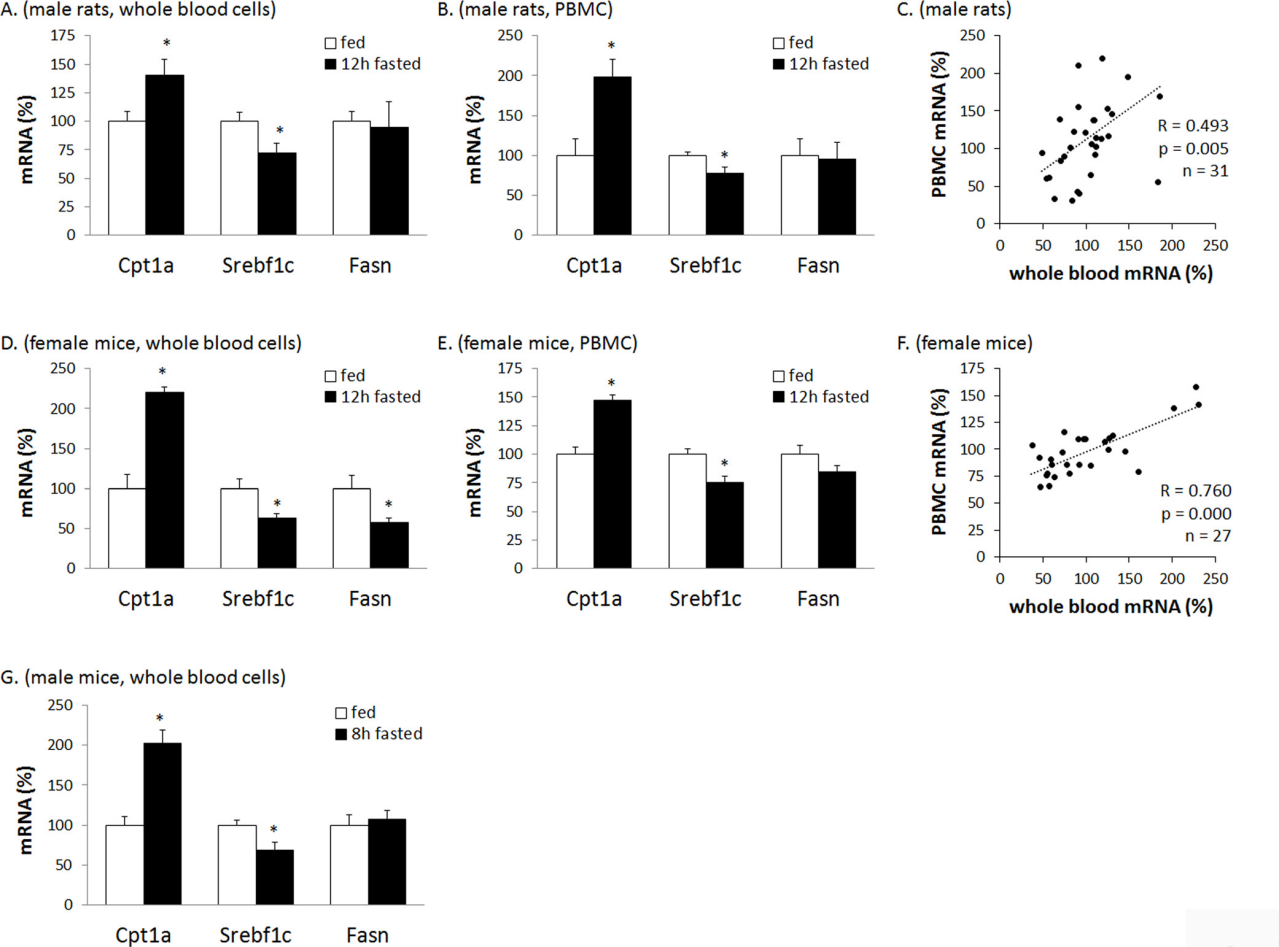
**Transcript responses to fasting in whole blood (A, D, G) and in PBMC (B, E) in rat and mice, and correlation between gene expression in whole blood and PBMC (C, F).** Data are the means±SEM of 5/6 animals per group. Male Wistar rats (A, B, C), female CD-1 mice (D, E, F) and male NMRI mice (G) were used. The expression levels of the indicated mRNAs were quantified by real-time RT-PCR, normalized to the expression of the housekeeping gene Actb, and expressed relative to the mean value of the respective fed group, which was set to 100. * indicates that mean values in the fasted group and the fed group are statistically different (P<0.05; Two-tailed Student’s *t* test). The value of the Pearson’s correlation index (R) and the level of significance (p) are given (C, E).

We have previously demonstrated that PBMC express key genes involved in energy homeostasis and can reflect transcriptional adaptations to acute changes in feeding conditions (fasting and refeeding) that occur in key tissues such as liver or adipose tissue [5–7, 22]. Here, we studied transcriptional responses to fasting in whole blood in rats and mice, and compared them to those in PBMC. We were able to quantify by real-time RT-PCR the mRNA levels of the key enzyme in FA beta-oxidation, carnitine palmitoyl-transferase 1a (Cpt1a), and of two genes related to FA synthesis, the sterol regulatory element-binding protein 1c gene (Srebf1c) and the FA synthase gene (Fasn), in whole blood RNA samples of rats and mice, with similar Ct values for these three genes in whole blood and PBMC. In accordance with the metabolic function of the encoded proteins, fasting consistently increased the expression of Cpt1a and decreased the expression of Srebf1c in whole blood RNA samples, both in rats and mice and (in the latter) irrespective of sex and anesthetics use (Fig 1A, 1D and 1G). Moreover, gene expression changes observed in whole blood RNA samples of fasted rats and mice followed the same pattern as in PBMC RNA samples of the same animals (Fig 1B and 1E) and the pattern previously described in PBMC of fasted rats [7, 22] and humans [4]. Fasting-induced down-regulation of Fasn expression [7] was better evidenced in mice after a 12h than an 8h-fast and in whole blood than in PBMC of 12h-fasted mice. Additionally, both in rats and mice there was a good correlation between whole blood and PBMC mRNA levels of the three genes studied (Fig 1C and 1F). Thus, we have shown that physiologically relevant transcriptional responses to fasting are reflected in the gene expression profile of whole blood as they are in the PBMC profile. These results underscore the great potential that whole blood gene expression profiling entails to detect changes in systemic metabolism or physiology in nutritional studies, which is of special interest when the amount of blood is limited, as happens in mouse studies.

### Whole blood can reflect transcriptional responses linked to HFD-induced overweight development as PBMC do

**Fig 2:**
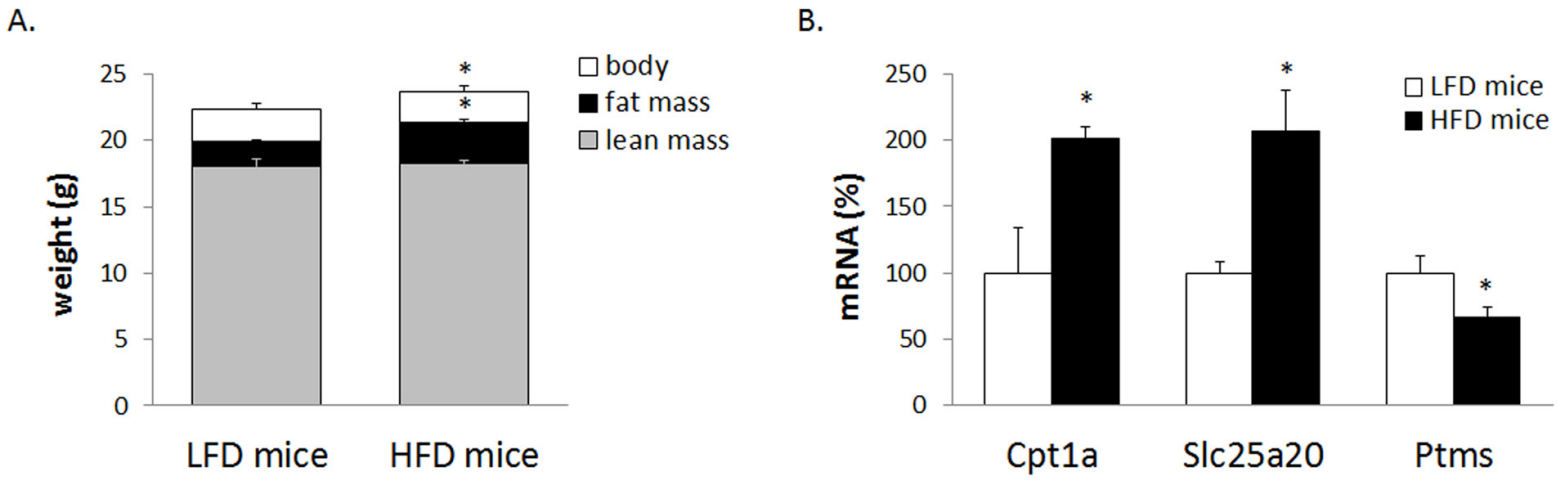
**Body composition (A) and transcript profile in whole blood (B) in response to HFD-induced overweight development in C57BL/6J male mice.** Data are the means±SEM of 5/6 animals per group. The expression levels of the indicated mRNAs were quantified by real-time RT-PCR, normalized to the expression of the housekeeping gene Actb, and expressed relative to the mean value of the control LFD group, which was set to 100. * indicates that mean values in the HFD group and the LFD group are statistically different (P<0.05; Two-tailed Student’s *t* test).

PBMC can reflect adaptations and deregulations related to increased adiposity induced by the intake of hyperlipidic diets [7, 12, 23] as well as metabolic recovery upon weight loss [22, 23]. Among top changes reported in PBMC transcriptome of diet-induced obese rats are an increased expression of Cpt1a and of mitochondrial carnitine/acylcarnitine translocase Slc25a20, as well as a decreased expression of parathymosin (Ptms) [12, 23]. These changes were reproduced in whole blood of obesity-prone mice fed a HFD for 21 days (Fig 2B), which attained a small but significant increase in both body weight and body fat content compared with LFD-fed control animals (Fig 2A). These results are in line with the idea that gene expression profile in whole blood can exhibit/reflect gene expression profiles linked to certain pathologies such as obesity and could be used for the monitoring of disease development in mouse models.

### Changes in gene expression in total blood of ATRA-treated mice reproduced known transcriptional effects of ATRA in liver and brown adipose tissue

Studies by independent groups including ours have shown that, in rodents, treatment with ATRA at different dosages and routes of administration reduces body weight and adiposity and enhances glucose tolerance and insulin sensitivity (see [24, 25]). ATRA-induced body weight and fat loss occurred despite unchanged [37–39] or even increased [40] energy intake and was accompanied by increases in body temperature [38, 40] and in the circulating levels of glycerol but not of NEFA [37, 38, 40]. These findings strongly suggested that the anti-adiposity action of ATRA arises from enhanced energy expenditure coupled to increased fat mobilization and oxidation of lipolysis-derived fatty acids. Indeed, the anti-adiposity action of ATRA in mice has been traced to decreased adipogenesis and adipokine production [41–44] along with increased oxidative metabolism in tissues including the liver [37], skeletal muscle [40, 45] and brown [46, 47] and white fat depots [38, 40, 42, 44, 48]. So, we set out to investigate whether whole blood gene expression could reflect well-studied ATRA effects in tissues of adult mice.

**Fig 3:**
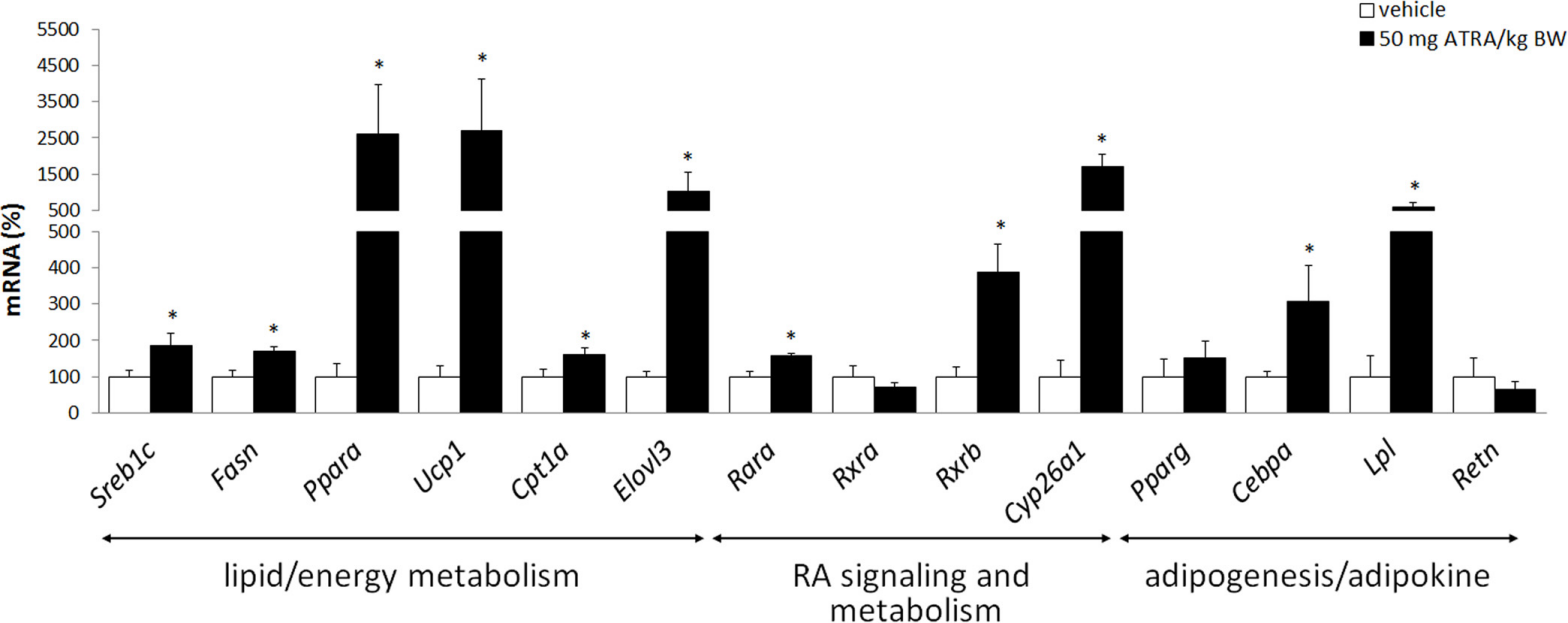
Transcript responses to ATRA treatment in whole blood. Data are the means±SEM of 5/6 mice per group. The expression levels of the indicated mRNAs were quantified by real-time RT-PCR, normalized to the expression of the housekeeping gene Actb, and aexpressed relative to the mean value of the control, vehicle-treated, group, which was set to 100. * indicates that mean values in ATRA- and vehicle-treated mice are statistically different (P<0.05; Two-tailed Student’s *t* test).

As shown in Table 2, ATRA treatment (50 mg/kg body weight/day during 4 consecutive days) led to a reduction in body weight and body fat without affecting energy intake, a decrease in blood glucose, a trend to reduced circulating NEFA and an increase in circulating β-hydroxybutyrate levels, in accordance with previous reports [37–39, 45]. When transcript-based responses to ATRA in whole blood of mice were studied, we observed a gene expression profile (Fig 3) that mimicked reported ATRA-induced changes in gene expression related to FA oxidation and lipogenesis in liver [37] and, especially, in cultured hepatocytes [49], namely an increased gene expression of peroxisome proliferator-activated receptor α (Ppara), Cpt1a, Srebf1c and Fasn. Moreover, gene expression in whole blood also reflected reported stimulatory effects of ATRA on BAT thermogenesis and WAT-to-BAT remodeling [38, 46–48, 50], as indicated by increased mRNA levels of uncoupling protein 1 (Ucp1), Ppara, Cpt1a and the ELOVL fatty acid elongase 3 (Elovl3) in whole blood of ATRA-treated compared with vehicle-treated mice (Fig 3). Nevertheless, reported ATRA effects inhibiting adipogenesis and adipokine expression in WAT and white adipocytes [42, 44, 51, 52] were not reflected, since the mRNA levels of the key adipogenic transcription factor CCAAT/enhancer binding protein α (Cebpa) and the Pparg responsive gene lipoprotein lipase (Lpl) were increased in whole blood of ATRA-treated compared with vehicle-treated mice, and the expression of the other key adipogenic transcription factor Pparg and the adipokine resistin (Retn) did not change (Fig 3).

**Table 2:** Body weight, food intake, blood glucose concentration, serum concentration of non-esterified fatty acids (NEFA) and β-hydroxybutyrate and weight of selected tissues in ATRA- and vehicle-treated mice.

		vehicle	50 mg ATRA/kg BW
body weight (g)		44.4±1.1	38.3±0.9*
food intake (g/kg BW)		496±37	429±34
glucose (mM)		9.07±0.71	6.82±0.51*
NEFA (mM)		1.32±0.11	0.98±0.14
3-OH-butyrate (mM)		0.56±0.14	0.94±0.09*
*tissue weights*			
	eWAT (g/100g BW)	2.15±0.26	1.53±0.12*
	liver (g/100g BW)	4.72±0.18	4.79±0.13
	gastrocnemius muscle (g/100g BW)	0.39±0.05	0.53±0.02*

Data are means ± SEM of 5–11 mice per group. Food intake represents the means ± SEM of 4 cages (2–3 animals each) per group, and was estimated from the actual amount of food consumed by the animals during 4 days-treatment.

* indicates that mean values in ATRA- and vehicle-treated mice are statistically different (P<0.05; Two-tailed Student’s *t* test).

Findings in this work indicate that gene expression in whole blood reflects known ATRA effects on FA and energy metabolism in adult mice, and suggest that gene expression in whole blood would mainly reflect or mimic the metabolic scenario found in densely vascularized tissues, like liver or BAT. Moreover, transcriptional markers (such as the Mest gene) of WAT expansion, a process tightly associated with angiogenesis [53], have been shown to be increased in whole blood of obesity-prone mice following short-term high fat diet feeding, during the active phase of body fat gain [21].

A direct effect of ATRA modulating gene expression in blood cells through their nuclear receptors (and/or other mechanisms) cannot be discarded. In fact, retinoic acid receptor (Rar) and retinoid X receptor (Rxr) isoforms were expressed in whole blood of mice (Fig 2), where they showed the same response to ATRA treatment that we have observed for these genes in BAT (Arreguin A, Ribot J, Bonet ML and Palou A, unpublished data). To be noted, RAR activation is key to ATRA-induced WAT-to-BAT remodeling [40, 48]. An involvement of direct ATRA effects in blood cells is further suggested by the strong upregulation of *bona fide* RAR target genes, such as the ATRA hydroxylase Cyp26a1 [54] and Ucp1 [55], found in whole blood of ATRA-treated mice (Fig 3), and by the fact that gene expression changes in blood more closely resembled changes in ATRA-treated hepatocytes in culture [49] than in liver of ATRA-treated mice [37]. Blood cells can directly respond to stimulus orchestrating complex transcriptional responses provided they express the involved regulatory factor(s); for instance, part of the fasting-induced changes in human PBMC result from direct activation of PPARα in these cells by free fatty acids (elevated in blood under fasting conditions), leading to an increment in the cells fat-handling capability, including increased capability for β-oxidation [4].

### Verification of potential transcript-based biomarkers of metabolic robustness in a mice genetic model of improved metabolic health using whole blood

Recently, Konieczna and collaborators [14] identified potential transcriptome-based early biomarkers of metabolic health related to lipid metabolism in PMBC of the offspring of dams submitted to 20% calorie restriction during lactation, which constitute a model of early priming of metabolic health, as these animals are protected against the development of dietary obesity and display other beneficial metabolic traits later in life compared to the progeny of *ad libitum* fed dams [56]. The proposed markers included an increased gene expression of Cpt1a, hormone-sensitive lipase (Lipe) and the steroidogenic acute regulatory protein (Star), and a decreased expression of Fasn, low density lipoprotein receptor-related protein 1 (Lrp1), Rxrb, sortilin-related receptor, LDLR class A repeats-containing (Sorl1) and phosphate cytidylyltransferase 2, ethanolamine (Pcyt2) in PBMC of calorie-restricted *versus* control young rats at weaning.

**Fig 4:**
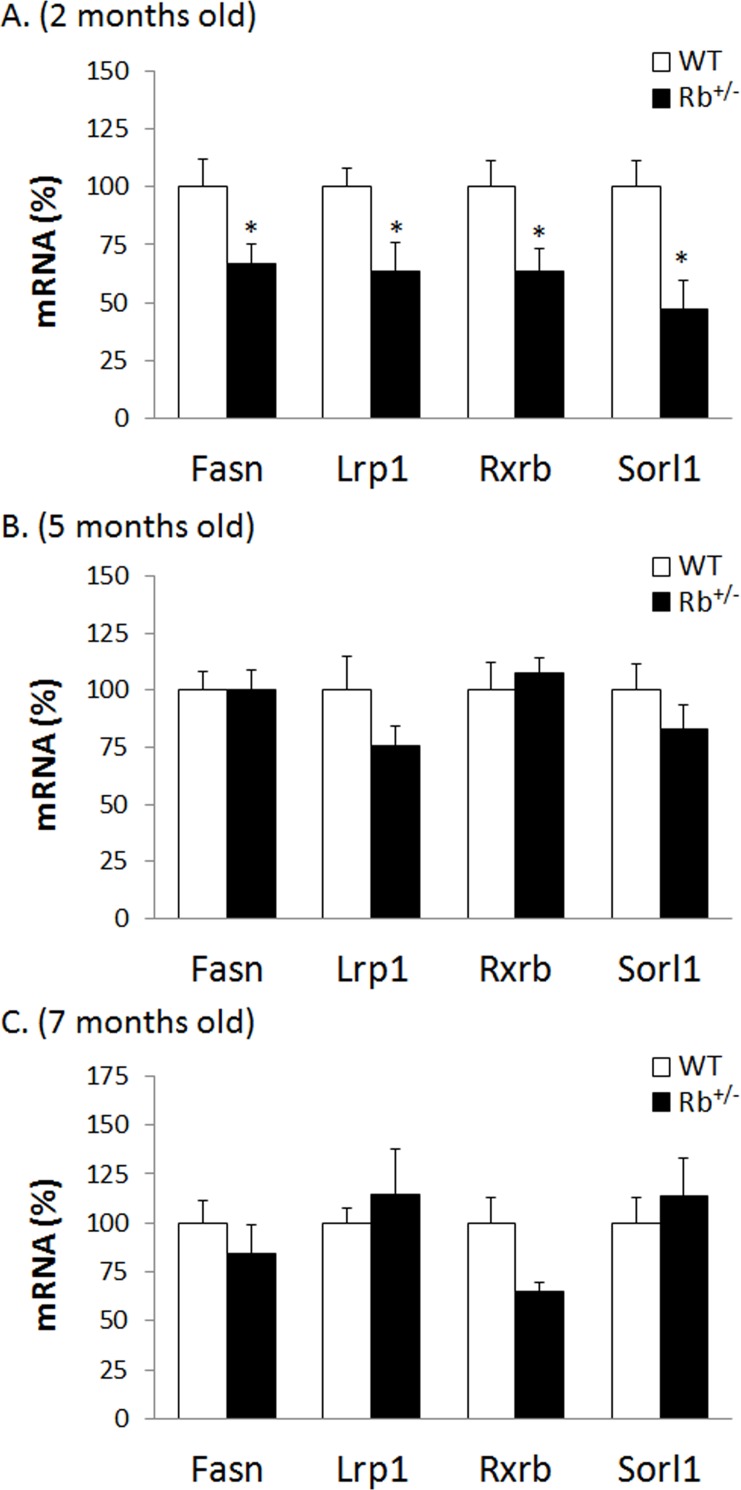
**Transcript levels of previously proposed putative biomarkers of health in whole blood of Rb**^**+/−**^ **and wild-type (WT) littermates at 2 months of age (A), 5 months of age (B) and 7 months of age (C).** Data are the means±SEM of 11/13 mice per genotype. The expression levels of the indicated mRNAs were quantified by real-time RT-PCR, normalized to the expression of the housekeeping gene Actb, and expressed, for each age, relative to the mean value of the WT littermates, which was set to 100. * indicates that mean values in Rb^+/−^ mice and WT littermates are statistically different (P<0.05; Two-tailed Student’s *t* test).

Early biomarkers of metabolic health, unlike the biomarkers derived from disease states, could serve to assess the potential benefits of bioactive compounds or changes in dietary habits aiming to decrease the risk of obesity and related metabolic alterations in healthy or at-risk individuals, and hence are of special interest in nutrition studies. Therefore, in this work we tackled this issue and aimed at verifying the aforementioned early transcript-based biomarkers in an unrelated model of improved metabolic health in mice and using whole blood RNA samples. The model chosen were mice with a partial germ-line deficiency in the Rb gene. We have previously shown that Rb^+/-^ mice are resistant to high fat diet-induced obesity [27] and that Rb haploinsufficiency provides advantages in front of acute metabolic stressors and ameliorates body fat gain and metabolic impairments that normally accompany the transition from young to mature adult age in standard diet-fed mice [26]. Here, Rb haploinsufficient mice and WT littermates were studied and characterized at 2-, 5- and 7-month of age (Table 3). Whereas no differences in body weight or composition were observed at young ages, mature adult Rb^+/–^mice were leaner than their WT littermates, displaying 26% reduced body fat content. Of the potential early biomarkers of metabolic health identified from PBMC transcriptome in rats, reduced expression of Fasn, Lrp1, Rxrb and Sorl1 were validated in 2-month-old Rb^+/–^mice using real-time RT-PCR on whole blood RNA (Fig 4A). At this young age, the Rb^+/–^mice had body weight and composition indistinguishable from WT mice but they already displayed signs of improved metabolic health which persisted through adulthood, when they also presented with reduced adiposity [26]. Star and Pcyt2 gene expression could not be reliable detected in mouse total blood RNA due to low expression levels, while Lipe and Cpt1a expressions were not significantly different in Rb^+/-^ and WT mice. We also examined the pattern of these lipid metabolism-related genes, which were significantly altered in the Rb^+/–^mice at the age of 2-month, in older animals (Fig 4B and 4C). As it happens in the rat model of metabolic health in which these biomarkers were originally identified [14], no significant changes in blood mRNA levels of Fasn, Lrp1, Rxrb and Sorl1 were observed between the healthier (now Rb^+/–^mice) and control (now WT littermates) groups at older ages, suggesting their potential usefulness as biomarkers at early ages only. Fasn, Lrp1, Rxrb and Sorl1 are involved in lipogenesis/adipogenesis and fat lipoprotein uptake (see [14]) and their downregulation might agree with protection against fat accumulation in Rb^+/–^mice compared with WT littermates. Therefore, using a complete different model and whole blood RNA, we have been able to validate reduced transcript levels of Fasn, Lrp1, Rxrb and Sorl1 in blood as early biomarkers of metabolic health which show potential to be used in the detection of changes in metabolism or physiology in mice studies, including studies in genetic mouse models.

**Table 3:** Body weight, body fat mass and body lean mass at 2, 5 and 7 months of age in Rb^+/−^ mice and wild-type (WT) littermates.

		WT	Rb^+/-^
*2-month old*			
	body weight (g)	24.0±0.53	24.7±0.72
	body fat mass (g)	2.90±0.10	2.94±0.06
	body lean mass (g)	19.1±0.54	19.6±0.68
*5-month old*			
	body weight (g)	29.8±0.74	30.3±1.31
	body fat mass (g)	4.99±0.44	3.96±0.18
	body lean mass (g)	22.4±0.58	23.9±1.36
*7-month old*			
	body weight (g)	30.3±0.60	30.5±1.57
	body fat mass (g)	4.85±0.38	3.60±0.15*
	body lean mass (g)	22.8±0.54	24.1±1.35

Data are means ± SEM of 4–8 mice per genotipe.

* indicates that mean values in Rb^+/−^ mice and WT littermates are statistically different (P<0.05; Two-tailed Student’s *t* test).

We wanted to ascertain to what extent gene expression changes in whole blood could reflect the metabolic environment and transcriptional changes in key tissues previously described in Rb^+/-^ mice. Because a prominent feature of the Rb^+/-^ mouse model is WAT-to-BAT remodeling (WAT browning) [26, 27], expression levels of selected genes related to lipid metabolism, thermogenesis, and the brite (brown in white) adipocyte phenotype were assessed in whole blood RNA samples of animals of different ages. We did not observe any significant differences in whole blood mRNA levels of Ppara, Pparg coactivator 1c (Pgc1c), Cpt1b, Ucp1 and the brite marker solute carrier family 27 (fatty acid transporter) member 1 (Slc27a1) between Rb^+/–^mice and WT littermates (data not shown), suggesting a poor potential of gene expression profiles of whole blood to reflect WAT browning in this model. To be noted, our recently published results indicate that bone morphogenetic protein (BMP) signaling pathways might be key to WAT browning in the Rb^+/-^ model [57] and mRNA expression levels in whole blood of most BMP receptors is very low, according to the web-based high performance search engine GENEVESTIGATOR^®^ platform (https://genevestigator.com/gv/index.jsp), unlike that of retinoid receptors (Rars and Rxrs) (GENEVESTIGATOR^®^ and this work). This fact could explain results obtained in this work evidencing that gene expression in whole blood of mice reflects WAT browning linked to ATRA treatment but not to Rb haploinsufficiency.

## Conclusion

Using several independent experimental paradigms, this study reveals the usefulness of whole blood transcriptional analysis as a tool in studies related to metabolic and physiological adaptations in front of acute metabolic stressors and nutritional interventions, as well as in the validation and assessment of early transcript-based biomarkers of metabolic health. This is of particular interest in studies using mouse models, where the amount of blood available is limited. Findings in this work also support reduced expression of Fasn, Lrp1, Rxrb and Sorl1 in blood cells at a young age as putative biomarkers of metabolic robustness.
